# Association of Daily Variance in Air Temperature With Postoperative Adhesive Small Bowel Obstruction

**DOI:** 10.7759/cureus.24176

**Published:** 2022-04-16

**Authors:** Yuta Yamamoto, Masato Kitazawa, Yusuke Miyagawa, Shigeo Tokumaru, Satoshi Nakamura, Makoto Koyama, Takehito Ehara, Nao Hondo, Yasuhiro Iijima, Yuji Soejima

**Affiliations:** 1 Surgery, Shinshu University School of Medicine, Matsumoto, JPN

**Keywords:** surgical management, daily variance, closed-loop obstruction, air temperature, adhesive small bowel obstruction

## Abstract

Introduction: The influence of air temperature on adhesive small bowel obstruction (ASBO) is unknown. This study aimed to investigate the relationship between air temperature and postoperative ASBO.

Methods: Overall, 312 patients with postoperative ASBO were included. They were categorized into two groups: the surgery group (n = 83) comprising patients who needed surgery, and the non-surgery group (n = 229) comprising patients who responded to conservative treatment. The associations between patients’ characteristics and weather variables on days of symptom onset with the need for surgical management were investigated. Weather variables included the daily mean barometric pressure, daily mean air temperature, day-to-day differences, daily variances, and diurnal variation in the air temperature. Day-to-day differences in weather variables were calculated as the daily mean variables on the day of symptom onset minus those on the previous day. The daily variances in weather variables were defined as the absolute value of day-to-day differences.

Results: Compared to the non-surgery group, the surgery group had older patients (75 vs. 70 years, p = 0.009), a higher proportion of female patients (44.6% vs. 32.3%, p = 0.046), increased incidence of closed-loop sign (50.6% vs. 0.9%, p < 0.001), a lower proportion of feces sign (18.1% vs. 31.3%, p = 0.024), and a prolonged hospital stay (11 vs. 22 days, p < 0.001). The number distribution of patients in the surgery group in day-to-day differences in air temperature was different from that of the non-surgery group; the former has several peaks whereas the latter has almost one peak. Daily variance in mean air temperature on the day of symptom onset was higher in the surgery group than in the non-surgery group (2.3 vs. 1.3℃, p < 0.001). Multiple logistic regression analysis revealed that increased daily variance in air temperature on the onset day was associated with the need for surgical management (odds ratio 1.254, p = 0.002) and closed-loop obstruction (odds ratio 1.235, p = 0.017). Regarding seasonal variations, the risk of the need for surgery and closed-loop obstruction in each ASBO patient was the highest in spring, followed by that in summer, autumn, and winter. Consistently, the daily variance in mean air temperature in spring was higher than that in summer, autumn, and winter (p < 0.0001, p < 0.0001, and p = 0.0047, respectively). The risk of the need for surgery and closed-loop obstruction in each ASBO patient was the highest in spring, followed by that in summer, autumn, and winter. Consistently, daily variance in mean air temperature was higher in spring than that in summer, autumn, and winter (p < 0.0001, p < 0.0001, and p = 0.0047, respectively).

Conclusion: Increased daily variance in mean air temperature on the day of onset is associated with the need for surgical management and closed-loop obstruction. Spring is characterized by the highest daily variance in mean air temperature among the four seasons, and is associated with high proportions of the need for surgery and closed-loop obstruction. These results can be clinically useful in terms of hospital resource reallocation and staffing, and can help clarify the pathogenesis of ASBO.

## Introduction

Adhesive small bowel obstruction (ASBO) is one of the most common complications that occur after intra-abdominal surgery. Several factors are associated with successful non-surgical management of patients with ASBO. For example, the feces sign (FS) [1-3], number of transitional zones [4], beak signs [5], anterior adhesion [6,7], sex differences [8], and administration of water-soluble contrast agents [6,7,9] affect the response of patients with ASBO to non-surgical management.

Recently, we reported that fluctuations in barometric pressure occurred in the pre-onset period in patients with postoperative ASBO, which indicates the impact of changes in weather variables on the onset of ASBO [10]. However, the influence of air temperature on ASBO has not yet been investigated.

The gastrointestinal tract is innervated by the enteric nervous system with cold chemosensors that are activated by low temperatures [11-13]. The enteric nervous system also influences secretory functions and modulates gastrointestinal tract motility [14,15]. Therefore, we hypothesized that air temperature could affect the etiology and the prognosis of patients with ASBO. This study aimed to investigate the relationship between air temperature and postoperative ASBO.

## Materials and methods

Patients and study design

**Figure 1:**
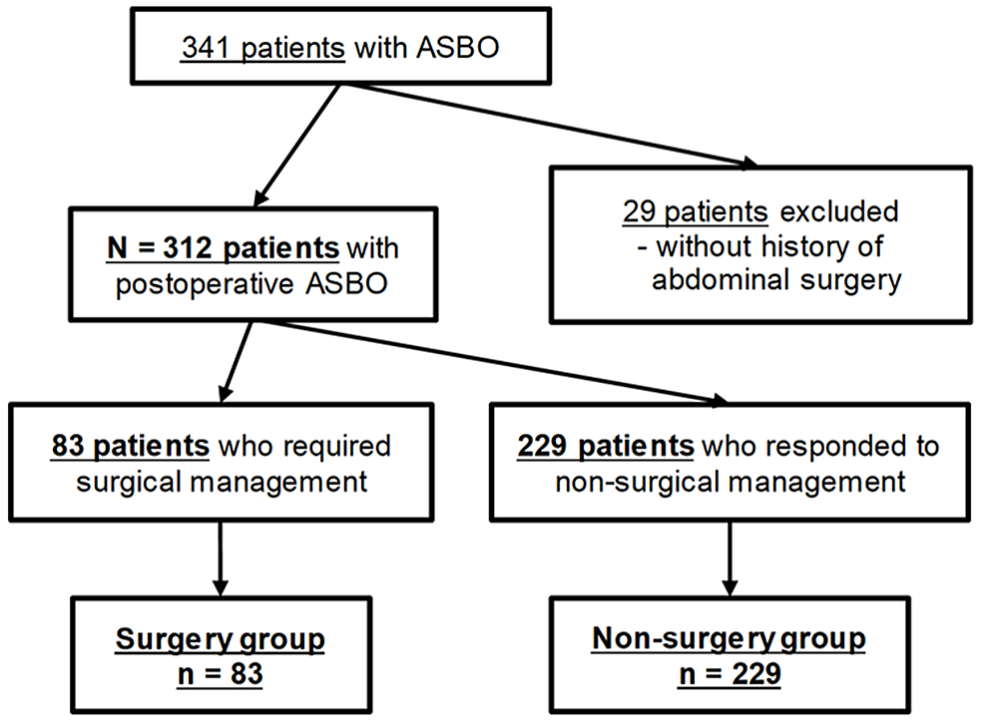
Flowchart of the selection of the study groups. ASBO, adhesive small bowel obstruction.

A total of 341 patients with ASBO were admitted to Shinshu University Hospital, Japan, between March 1, 2007 and April 30, 2021. ASBO was diagnosed based on the presence of two criteria: 1) clinical symptoms, including nausea, vomiting, and abdominal pain; and 2) radiological imaging that showed a dilated small intestine with a diameter >2.5 cm. We excluded 29 patients who did not undergo abdominal surgery before the onset of ASBO. Our final study group comprised 312 patients with postoperative ASBO. We retrospectively reviewed their medical records and categorized them into two groups: the surgery group (n = 83), which comprised those who needed surgery, and the non-surgery group (n = 229), which comprised those who responded to conservative treatment (shown in Figure 1). Furthermore, we assessed the daily mean barometric pressure and the daily mean air temperature and determined their day-to-day differences and daily variances. Day-to-day differences in weather variables were calculated as the daily mean variables on the day of symptom onset minus those on the previous day. The daily variances in weather variables were defined as the absolute values of day-to-day differences. The daily variances in air temperature in the peri-onset period were also defined as the absolute differences between the daily mean air temperature on the index day and their respective values on the previous day. Diurnal variation in air temperature was defined as the variation between the highest and the lowest air temperature that occurred during the day of onset. The Shinshu University Hospital is located in Matsumoto City (altitude, 610 m), almost at the center of the main island of Japan. Daily weather variables in Matsumoto City were measured using the Automated Meteorological Data Acquisition System at the Matsumoto Meteorological Station, and the website of the Japan Meteorological Agency (URL: www.jma.go.jp/jma/index.html). Barometric pressure and air temperature were recorded as the average of continuous measurements from 0:00 to 24:00. The associations between the patients’ characteristics, need for surgical management, closed-loop obstruction and weather variables were investigated.

Regarding the management of ASBO, intravenous fluids were administered to all patients. Initially, we assessed the presence of strangulation of the small intestine, which requires emergency surgery. When strangulation was excluded, the patients with non-strangulated ASBO were considered as candidates for non-surgical management. Regarding the general treatment strategies for non-strangulated ASBO, patients with active symptoms, such as abdominal pain and nausea, were treated using gastrointestinal decompression methods, including nasogastric tube placement and long tube placement. If intestinal obstruction continued for more than seven days, or if it recurred after the commencement of oral feeding, non-surgical management was considered to have failed and the patients underwent surgery. A closed-loop sign was defined as an obstruction of two points of the small intestine at the same site, and this was confirmed using computed tomography (CT) imaging.

Lastly, we analyzed the seasonal variations in the incidence of ASBO, risk of the need for surgery, proportion of the closed-loop obstruction, and daily variance in mean air temperature during the study period. Four seasons were defined as follows: spring, March 1 to May 31; summer, June 1 to August 31; autumn, September 1 to November 30; and winter, December 1 to February 28.

Statistical analysis

Demographic data were presented using descriptive statistics. Continuous variables were presented as medians with interquartile ranges (IQR) and were compared using the Mann-Whitney test. Multiple logistic regression analysis was performed to identify factors that were independently associated with the need for surgical management, emergency surgery, and closed-loop obstruction. The results of multiple logistic regression analysis are presented as odds ratios (ORs) with 95% confidence intervals (CIs). Unpaired t-test with Bonferroni correction for multiple comparison was used to compare daily variance in mean air temperature between the four seasons. In this test, a p-value of <0.0125 (0.05÷4) was considered statistically significant. All the other tests were two-tailed, and, except for unpaired t-test with Bonferroni correction, a p-value of <0.05 was considered statistically significant. Statistical analyses were performed using the Statistical Package for Social Sciences version 23.0 (IBM Corp., Armonk, NY, USA).

## Results

The patients’ characteristics are presented in Table 1. The median age of the patients was 71 years (IQR, 62-79 years), and 201 patients (64.4%) were male. Two hundred and sixteen patients (69.2%) had undergone a prior surgery due to an abdominal malignancy as the primary disease. Two hundred and eighty-two patients (90.4%) had a good performance status (PS; Eastern Cooperative Oncology Group PS 0 and 1). Overall, 83 patients (26.6%) underwent surgical management, and 49 patients (15.7%) underwent surgery within 48 hours of hospital admission.

**Table 1:** Patients’ characteristics ^a^Other malignancy includes hypopharyngeal cancer (six patients), liver cancer (two patients), biliary cancer (two patients), and liposarcoma (two patients). ASBO, adhesive small bowel obstruction; CT, computed tomography; IQR, interquartile range.

Variables			Total (n = 312)
Sex			
	Male (%)		201 (64.4)
	Female (%)		111 (35.6)
Age (years)			
	Median (IQR)		71 (62–79)
Body mass index (kg/m^2^)			
	Median (IQR)		19.6 (18.0–22.0)
Primary disease			
	Benign (%)		96 (30.8)
	Malignancy (%)		216 (69.2)
		Esophagus/Stomach (%)	65 (20.8)
		Colon/Rectum (%)	74 (23.7)
		Uterus/Ovary (%)	49 (15.7)
		Bladder/Urinary tract (%)	16 (5.1)
		Other (%)^a^	12 (3.8)
Performance Status			
	0,1 (%)		282 (90.4)
	2–4 (%)		30 (9.6)
CT findings			
	Feces sign (%)		86 (27.6)
	Closed-loop sign (%)		44 (14.1)
Treatment of ASBO			
	Non-surgical management (%)		229 (73.4)
	Surgical management (%)		83 (26.6)
		Emergency surgery (within 48hours after admission)	49 (15.7)
		Non-emergency surgery	34 (10.9)

Compared to the non-surgery group, the surgery group had older patients (75 years vs. 70 years, p = 0.009), a higher proportion of female patients (44.6% vs. 32.3%, p = 0.046) and increased incidence of closed-loop sign (50.6% vs. 0.9%, p < 0.001), a lower proportion of patients with the feces sign (18.1% vs. 31.3%, p = 0.024), and had prolonged length of hospital stay (11 days vs. 22 days, p < 0.001). Regarding weather variables, the daily variance in mean air temperature on the day of symptom onset in the surgery group was larger than that in the non-surgery group (2.3 ℃ vs. 1.3 ℃, p < 0.001), whereas there was no difference in the diurnal variation in air temperature between these groups (11.0 ℃ vs. 10.3 ℃, p = 0.140) (Table 2).

**Table 2:** Comparison of demographics of patients between surgery and non-surgery group *A statistical significance (p < 0.05). hPa, hectopascal; IQR, interquartile range.

Demographic Characteristics			Surgery (n = 83)	Non-surgery (n = 229)	P-value
Age (year)					
	Median (IQR)		75 (65–81)	70 (62–77)	0.009*
Sex					
	Male (%)		46 (55.4)	155 (67.7)	0.046*
	Female (%)		37 (44.6)	74 (32.3)	
Performance status					
	1,2 (%)		74 (89.2)	208 (90.8)	0.658
	2–4 (%)		9 (10.8)	21 (9.2)	
Body mass index (kg/m^2^)					
	Median (IQR)		19.5 (17.1–21.6)	19.6 (18.1–22.2)	0.089
Primary disease					
	Benign (%)		29 (34.9)	67 (29.3)	0.337
	Esophageal/Gastric cancer (%)		13 (15.7)	52 (22.7)	0.176
	Colorectal cancer (%)		20 (24.1)	54 (23.6)	0.925
	Other cancer (%)		21 (25.3)	56 (24.5)	0.878
Postoperative period (>5 years)					
	>5 years (%)		39 (47.0)	116 (50.7)	0.567
	≤5 years (%)		44 (53.0)	113 (49.3)	
Surgical approach for primary disease					
	Laparoscopy (%)		4 (4.8)	9 (3.9)	0.728
	Laparotomy (%)		79 (95.2)	220 (96.1)	
History of chemotherapy					
	Yes (%)		15 (18.1)	60 (26.2)	0.138
	No (%)		68 (81.9)	169 (73.8)	
History of radiotherapy					
	Yes (%)		3 (3.6)	15 (6.6)	0.326
	No (%)		80 (96.4)	214 (93.4)	
White blood cell count (/µl)					
	Median (IQR)		8600 (6750–11430)	8430 (6695–10795)	0.889
Feces sign					
	Positive (%)		15 (18.1)	71 (31.0)	0.024*
Closed-loop sign					
	Positive (%)		42 (50.6)	2 (0.9)	<0.001*
Length of hospital stay (day)					
	Median (IQR)		11 (8–16)	22 (12–33)	<0.001*
Weather variables					
	Mean barometric pressure (hPa)				
		Median (IQR)	945.1 (940.6–947.6)	943.5 (939.6–948.1)	0.587
	Day-to-day differences in mean barometric pressure (hPa)				
		Median (IQR)	-1.1 (-3.6–2.6)	0.4 (-3.0–3.3)	0.315
	Daily variance in mean barometric pressure (hPa)				
		Median (IQR)	3.3 (1.5–5.4)	3.2 (1.3–5.5)	0.633
	Mean air temperature (℃)				
		Median (IQR)	12.5 (4.7–20.9)	11.9 (4.2–19.4)	0.367
	Day-to-day differences in mean air temperature (hPa)				
		Median (IQR)	-0.1 (-2.5–1.6)	0 (-1.4–1.3)	0.514
	Daily variance in mean air temperature (℃)				
		Median (IQR)	2.3 (1.1–3.3)	1.3 (0.5–2.4)	<0.001*
	Diurnal variation in air temperature (℃)				
		Median (IQR)	11.0 (8.6–13.6)	10.3 (8.1–12.9)	0.140

**Figure 2:**
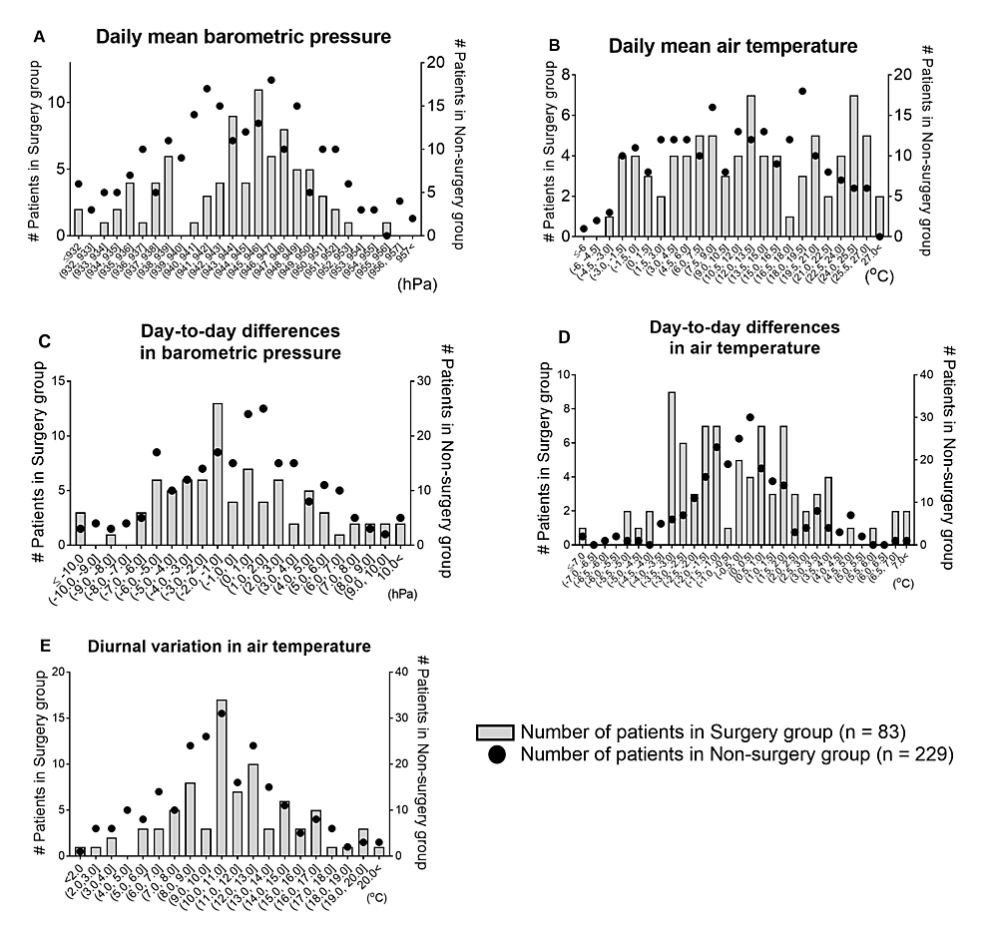
Distribution of the number of patients in the surgery group (n = 83) and in the non-surgery group (n = 229), for each of the five weather variables (A-E) (A) Daily mean barometric pressure; (B) daily mean air temperature; (C) day-to-day differences in barometric pressure; (D) day-to-day differences in air temperature; (E) diurnal variation in air temperature.

Regarding weather variables, Figures 2A-2E show the distributions of the number of patients in the surgery and non-surgery groups for each of the five weather variables. The shapes of the histograms for the number of patients in the surgery group relative to day-to-day differences in air temperature are quite different from that in the non-surgery group, as the former has several peaks whereas the latter has nearly one peak. In this context, the daily variance in mean air temperature on the day of symptom onset in the surgery group was larger than that in the non-surgery group (2.3℃ vs. 1.3℃, p < 0.001) (Table 2).

**Figure 3:**
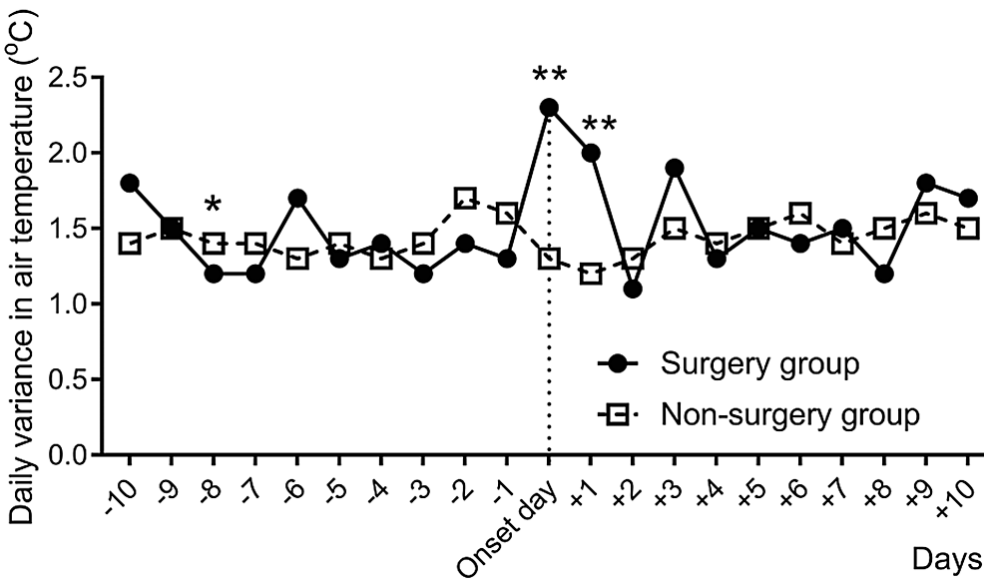
Changes in the daily variances in air temperature in each group over time during the peri-onset period. * indicates p < 0.05. ** indicates p < 0.001.

Figure 3 and Table 3 show the daily variance in mean air temperature in the peri-onset period. The variance in the surgery group increased suddenly on the day of onset, resulting in a statistically significant difference between the variances on the day of onset and those on post-onset day one, compared to those in the non-surgery group (2.3℃ vs. 1.3℃, p < 0.001 and 2.0℃ vs. 1.2℃, p < 0.001, respectively).

**Table 3:** Comparison of daily variances in mean air temperature between surgery and non-surgery groups *A statistical significance (p < 0.05). hPa, hectopascal; IQR, interquartile range.

		Surgery (n = 83)	Non-surgery (n = 229)	P-value
Pre-onset day 10 (°C)				
	Median (IQR)	1.8 (0.7–2.9)	1.4 (0.7–2.6)	0.192
Pre-onset day 9 (°C)				
	Median (IQR)	1.5 (0.6–2.4)	1.5 (0.6–2.5)	0.552
Pre-onset day 8 (°C)				
	Median (IQR)	1.2 (0.5–1.9)	1.4 (0.6–2.8)	0.049*
Pre-onset day 7 (°C)				
	Median (IQR)	1.2 (0.6–2.4)	1.4 (0.6–2.6)	0.406
Pre-onset day 6 (°C)				
	Median (IQR)	1.7 (0.8–2.7)	1.3 (0.6–2.6)	0.113
Pre-onset day 5 (°C)				
	Median (IQR)	1.3 (0.6–2.4)	1.4 (0.5–2.7)	0.247
Pre-onset day 4 (°C)				
	Median (IQR)	1.4 (0.8–2.4)	1.3 (0.7–2.3)	0.675
Pre-onset day 3 (°C)				
	Median (IQR)	1.2 (0.4–2.5)	1.4 (0.7–2.9)	0.121
Pre-onset day 2 (°C)				
	Median (IQR)	1.4 (0.7–2.8)	1.7 (0.8–2.9)	0.423
Pre-onset day 1 (°C)				
	Median (IQR)	1.3 (0.5–3.5)	1.6 (0.8–2.6)	0.728
Onset day (°C)				
	Median (IQR)	2.3 (1.1–3.4)	1.3 (0.5–2.4)	<0.001*
Post-onset day 1 (°C)				
	Median (IQR)	2.0 (1.2–3.6)	1.2 (0.6–2.8)	<0.001*
Post-onset day 2 (°C)				
	Median (IQR)	1.1 (0.6–2.5)	1.3 (0.6–2.5)	0.609
Post-onset day 3 (°C)				
	Median (IQR)	1.9 (0.7–3.1)	1.5 (0.8–2.8)	0.244
Post-onset day 4 (°C)				
	Median (IQR)	1.3 (0.6–3.0)	1.4 (0.7–2.6)	0.854
Post-onset day 5 (°C)				
	Median (IQR)	1.5 (0.6–2.4)	1.5 (0.6–2.8)	0.988
Post-onset day 6 (°C)				
	Median (IQR)	1.4 (0.6–2.6)	1.6 (0.6–2.7)	0.787
Post-onset day 7 (°C)				
	Median (IQR)	1.5 (0.5–2.6)	1.4 (0.5–2.6)	0.941
Post-onset day 8 (°C)				
	Median (IQR)	1.2 (0.6–2.5)	1.5 (0.7–2.5)	0.359
Post-onset day 9 (°C)				
	Median (IQR)	1.8 (0.9–2.6)	1.6 (0.6–2.8)	0.824
Post-onset day 10 (°C)				
	Median (IQR)	1.7 (0.7–2.9)	1.5 (0.6–2.7)	0.404

Multiple logistic regression analyses revealed that increased daily variance in the mean air temperature on the day of onset, as well as the absence of the feces sign, were associated with the need for surgical management (OR 1.254, 95% CI 1.086-1.447, p = 0.002, and OR 0.513, 95% CI 0.271-0.972, p = 0.041) (Table 4). Moreover, increased daily variance in the air temperature was associated with the presence of closed-loop obstruction (OR 1.235, 95% CI 1.039-1.467, p = 0.017) (Table 5).

**Table 4:** Multiple logistic regression analysis of surgical treatment *A statistical significance (p < 0.05). CI, confidence interval.

Variables	Univariate			Multivariate		
	Odds ratio	95% CI	P-value	Odds ratio	95% CI	P-value
Age (per 1 year increase)	1.016	0.996–1.035	0.111			
Female sex (reference group: male sex)	1.685	1.008–2.186	0.047*	1.669	0.984–2.832	0.057
Performance status (2–4) (reference group: performance status (0, 1))	1.205	0.528–2.748	0.658			
Body mass index (per 1 kg/m^2^ increase)	0.929	0.860–1.003	0.061			
Primary disease (benign) (reference group: malignant diseases)	1.299	0.762–2.214	0.337			
Primary disease (esophageal/gastric cancer) (reference group: diseases except esophageal/gastric cancer)	0.632	0.324–1.233	0.178			
Primary disease (colorectal cancer) (reference group: diseases except colorectal cancer)	1.029	0.571–1.853	0.925			
Primary disease (other cancer) (reference group: diseases except colorectal cancer)	1.046	0.586–1.868	0.878			
Postoperative period (>5 years) (reference group: postoperative period ≤5 yers)	0.863	0.522–1.428	0.567			
Surgical approach for primary disease (laparoscopy) (reference group: laparotomy)	1.238	0.371–4.132	0.729			
History of chemotherapy (reference group: no history of chemotherapy)	0.621	0.330–1.169	0.140			
History of radiotherapy (reference group: no history of radiotherapy)	0.535	0.151–1.897	0.333			
White blood cell count (per 1/μL increase)	1.000	1.000–1.000	0.849			
Feces sign (reference group: feces sign negative)	0.491	0.263–0.917	0.026*	0.513	0.271–0.972	0.041*
Mean barometric pressure (per 1 hPa increase)	1.008	0.966–1.051	0.718			
Day-to-day differences in mean barometric pressure (per 1 hPa increase)	0.978	0.927–1.031	0.410			
Daily variance in mean barometric pressure (per 1 hPa increase)	1.034	0.950–1.127	0.437			
Mean air temperature (per 1 °C increase)	1.013	0.985–1.043	0.360			
Day-to-day differences in mean air temperature (per 1 °C increase)	0.993	0.901–1.095	0.891			
Daily variance in mean air temperature (per 1 °C increase)	1.262	1.096–1.454	0.001*	1.254	1.086–1.447	0.002*
Diurnal variation in air temperature (per 1 °C increase)	1.050	0.986–1.119	0.130			

**Table 5:** Multiple logistic regression analysis of closed-loop obstruction *A statistical significance (p < 0.05). CI, confidence interval.

Variables	Univariate			Multivariate		
	Odds ratio	95% CI	P-value	Odds ratio	95% CI	P-value
Age (per 1 year increase)	1.049	1.018–1.081	0.002*	1.049	1.018–1.081	0.002*
Female sex (reference group: male sex)	1.621	0.850–3.089	0.142			
Performance status (2–4) (reference group: performance status (0, 1))	2.015	0.808–5.026	0.133			
Body mass index (per 1 kg/m^2^ increase)	0.978	0.892–1.073	0.638			
Primary disease (benign) (reference group: malignant diseases)	1.059	0.533–2.101	0.871			
Primary disease (esophageal/gastric cancer) (reference group: diseases except esophageal/gastric cancer)	0.444	0.168–1.177	0.103			
Primary disease (colorectal cancer) (reference group: diseases except colorectal cancer)	1.423	0.701–2.888	0.329			
Primary disease (other cancer) (reference group: diseases except colorectal cancer)	1.171	0.570–2.406	0.667			
Postoperative period (>5 years) (reference group: postoperative period ≤5 yers)	1.397	0.734–2.656	0.308			
Surgical approach for primary disease (laparoscopy) (reference group: laparotomy)	2.878	0.846–9.786	0.091			
History of chemotherapy (reference group: no history of chemotherapy)	0.667	0.295–1.505	0.329			
History of radiotherapy (reference group: no history of radiotherapy)	0.343	0.045–2.647	0.305			
White blood cell count (per 1 /μL increase)	1.000	1.000–1.000	0.474			
Feces sign (reference group: feces sign negative)	0.638	0.293–1.390	0.258			
Mean barometric pressure (per 1 hPa increase)	0.988	0.936–1.042	0.647			
Day-to-day differences in mean barometric pressure (per 1 hPa increase)	0.980	0.916–1.048	0.560			
Daily variance in mean barometric pressure (per 1 hPa increase)	0.985	0.881–1.102	0.792			
Mean air temperature (per 1 °C increase)	1.037	0.999–1.076	0.056			
Day-to-day differences in mean air temperature (per 1 °C increase)	0.969	0.856–1.098	0.622			
Daily variance in mean air temperature (per 1 °C increase)	1.214	1.031–1.430	0.020*	1.235	1.039–1.467	0.017*
Diurnal variation in air temperature (per 1 °C increase)	1.050	0.970–1.138	0.229			

**Figure 4:**
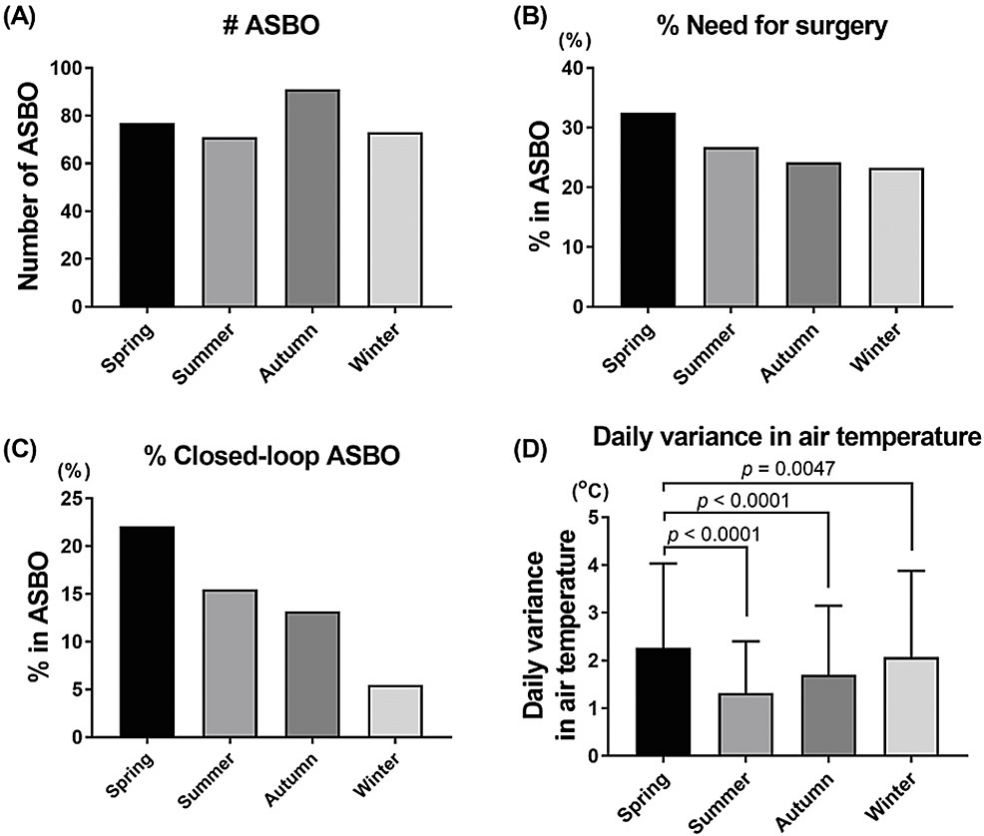
Seasonal variations in adhesive small bowel obstruction and daily variance in mean air temperature Each graph shows seasonal variations in the incidence of ASBO (A), proportion of patients needing surgery (B), proportion of patients with closed-loop obstruction (C), and daily variance in mean air temperature (D). Data are presented as mean and standard deviation (D). P-values were determined by a two-tailed unpaired t-test with Bonferroni correction for multiple comparisons, and a p-value of <0.0125 was considered statistically significant. ASBO, adhesive small bowel obstruction.

Figure 4 shows the associations between seasons and the incidence of ASBO, the risk of the need for surgery, and the proportion of the closed-loop obstruction. The incidence of ASBO was higher in autumn than in any other season (Figure 4A). On the other hand, the risk of the need for surgery and closed-loop obstruction in each ASBO patient was the highest in spring, followed by that in summer, autumn, and winter (Figures 4B, 4C). Consistently, daily variance in mean air temperature in spring was higher than that in summer, autumn, and winter (p < 0.0001, p < 0.0001, and p = 0.0047, respectively) (Figure 4D).

## Discussion

In the present study, we report that increased daily variance in mean air temperature on the day of symptom onset is associated with the need for surgery and the presence of a closed-loop obstruction in patients with ASBO. Our findings can predict the need for surgery based on the daily variance in mean air temperature, which is clinically useful for the reallocation of hospital resources and staffing, as well as help clarify the pathogenesis of ASBO.

The gastrointestinal tract is directly associated with temperature. The gastrointestinal tract is innervated by the enteric nervous system, comprising a widespread neural network enveloped by the intestinal wall, and controls motility, peristalsis, and intestinal secretions [16]. The transient receptor potential ion channel families are expressed by primary afferent neurons operating as cold chemosensors and activated by cold temperature in the gastrointestinal tract [11-13]. These neurons are involved in secretory mechanisms and modulation of the gastrointestinal tract motility [14,15], suggesting that fluctuations in air temperature affect the small intestine and may contribute to the pathogenesis of ASBO. A closed-loop obstruction occurs when two points of the small intestine are obstructed at the same site, and requires immediate surgical intervention [17,18]. It is observed in patients with ASBO due to an adhesive band and is not seen in patients with ASBO due to a matted adhesion [19]. These reports indicate the different etiologies of ASBO with and without a closed-loop sign. Our results suggest that the change in intestinal motility and secretion caused by fluctuation in air temperature can accelerate the onset of a closed-loop obstruction, which is associated with adhesive bands. This does not mean that daily variance of air temperature leads to deterioration in the condition of patients with ASBO but rather means that such variance is associated with the incidence of ASBO that requires surgical intervention, such as closed-loop obstruction. In the multiple logistic regression analysis, the closed-loop sign was not used as a predictor variable of surgical management. This is because the sign has already been reported to be associated with the requirement for immediate surgical intervention [17,18] and was regarded as an intermediate variable.

Regarding seasonal variations (Figure 4), although not statistically proven, spring is associated with a high risk of the need for surgery and closed-loop obstruction. Consistently, the daily variance in mean air temperature in spring was the highest among all seasons. These results support our data that increased daily variance in mean air temperature on the day of symptom onset of ASBO is associated with the need for surgery and the presence of a closed-loop obstruction.

This study has several limitations. First, the daily variance in mean air temperature in our result seemed too small to affect the small intestine. Diurnal variations in air temperature on the onset day in both groups were more than 10 ℃, thus, the value of the daily variance in mean air temperature on the onset day was considerably lower than the change in air temperature that patients with ASBO were exposed on onset day. Therefore, the daily variance in air temperature may be a surrogate marker of a meteorological factor that increased the incidence of ASBO requiring surgical management. Second, the weather variables we used did not perfectly represent the exact values at the specific times and locations of patients with ASBO. This is because not all the patients in this study lived in Matsumoto City, and the weather variables that we used in this study were the mean values in 24 hours. Finally, this was a single-center retrospective study and our findings may be subject to selection bias. Therefore, prospective multi-center studies that include a larger patient population are necessary to confirm these findings.

## Conclusions

We report that increased daily variance in mean air temperature on the day of symptom onset is associated with the need for surgery in patients with ASBO. In addition, it is related to the occurrence of closed-loop obstruction. Further, spring, characterized by the highest daily variance in mean air temperature among four seasons, is associated with high proportions of the need for surgery and closed-loop obstruction.

ASBO is a common cause of postoperative morbidity and can be a life-threatening disease. These results can be clinically useful in terms of reallocation of hospital resources and staffing, as well as clarification of the pathogenesis of ASBO.
